# Application of A Novel Potential Probiotic *Lactobacillus paracasei* Strain Isolated from Kefir Grains in the Production of Feta-Type Cheese

**DOI:** 10.3390/microorganisms6040121

**Published:** 2018-11-29

**Authors:** Ioanna Mantzourani, Antonia Terpou, Athanasios Alexopoulos, Pelagia Chondrou, Alex Galanis, Argyro Bekatorou, Eugenia Bezirtzoglou, Athanasios A Koutinas, Stavros Plessas

**Affiliations:** 1 Laboratory of Microbiology, Biotechnology and Hygiene, Faculty of Agricultural Development, Democritus University of Thrace, 68200 Orestiada, Greece; alexopo@agro.duth.gr (A.A.); empezirt@agro.duth.gr (E.B.); splessas@agro.duth.gr (S.P.); 2 Department of Chemistry, Food Biotechnology, University of Patras, 26500 Patras, Greece; aterpou@upatras.gr (A.T.); abekatorou@upatras.gr (A.B.); a.a.koutinas@upatras.gr (A.A.K.); 3 Department of Molecular Biology and Genetics, Democritus University of Thrace, 68100 Alexandroupolis, Greece; pelagiaho@gmail.com (P.C.); agalanis@mbg.duth.gr (A.G.)

**Keywords:** Kefir, Feta-type cheese, *Lactobacillus*, probiotic potential, *L. paracasei* SP3

## Abstract

In the present study 38 lactic acid bacteria strains were isolated from kefir grains and were monitored regarding probiotic properties in a series of established in vitro tests, including resistance to low pH, resistance to pepsin and pancreatin, and tolerance to bile salts, as well as susceptibility against common antibiotics. Among them, the strain SP3 displayed potential probiotic properties. Multiplex PCR analysis indicated that the novel strain belongs to the paracasei species. Likewise, the novel strain (*Lactobacillus paracasei* SP3) was applied as a starter culture for Feta-type cheese production. Feta-type cheese production resulted in significantly higher acidity; lower pH; reduced counts of coliforms, yeasts and fungi; and improved quality characteristics compared with cheese samples produced with no starter culture. Finally, it is highlighted that the application of the novel strain led to Feta-type cheese production with improved overall quality and sensory characteristics.

## 1. Introduction

The last decade, growing attention has been devoted to the expansion of functional foods produced by incorporated probiotic microorganisms [1,2]. Probiotics are defined as viable microorganisms which can exert various beneficial effects to the host when ingested at an appropriate concentration [3,4]. Among their many beneficial effects, probiotics have been proposed to provide anti-pathogenic, anti-carcinogenic, and anti-mutagenic activities in addition to cholesterol removal and alleviation of lactose intolerance [3,5,6,7]. Among the known probiotic microorganisms, species of lactic acid bacteria (LAB) have a long history of safe use [8]. Furthermore, the metabolic products of LAB have been proposed to demonstrate a positive effect on flavor, texture, and enhanced shelf-life of fermented food products [9]. Likewise, many LAB with probiotic characteristics are frequently used in fermented products like, yogurts [10,11], cheese [12,13], bread [14,15], meat products [16], and various beverages [17,18], etc., providing food with enhanced organoleptic, nutritional, and health-promoting attributes. Recently, scientific research has been dedicated to the selection and characterization of probiotic strains for food production targeting enhanced viability and exert functional proprieties [19]. As a result scientific research has recently focused on the isolation of novel probiotic strains’ origins from traditional food products, like kefir grains [20], cheese [21], fermented olives [22], meat products [23], fermented fish [24], etc., aiming to investigate possible novel properties and characteristics by their application in food production.

To express their beneficial effects on the host, newly isolated microorganisms must survive through the gastric transit and colonize the intestinal tract of the consumer; they should also present probiotic characteristics such as auto-aggregation and co-aggregation ability, and antagonistic activity against pathogens. Furthermore, antibiotic resistance of novel potential probiotics needs to be monitored due to the potential health risk that might occur by antibiotic resistance genes transferred from probiotic bacteria into the resident microbiota of the hosts gastrointestinal (GI) tract and, hence, to pathogenic bacteria [25]. Another factor that directly influences probiotics beneficial effect is their viability within the products matrix during food manufacture, distribution, and storage. For this to occur, probiotic food products need to provide at least 10^6^ CFU/g live probiotic microorganisms [2,4].

Cheese has been characterized as a suitable food matrix for the delivery of probiotics while recently many studies have highlighted the good impact of probiotics addition on cheese physicochemical, microbiological, and sensory characteristics [12,13,26]. Greece has a long tradition in the production of several dairy products, with Feta cheese being the most dominant one [27].

The targets of the present study were: (i) the isolation of novel LAB strains from kefir grains, (ii) the assessment of their probiotic potential through a series of established in vitro tests, (iii) the evaluation of their susceptibility against common antibiotics (iv) the molecular characterization of the isolate with the most anticipated probiotic properties, and (v) the study of its technological properties as starter culture in Feta cheese production.

## 2. Materials and Methods

### 2.1. Kefir Grains Production

Kefir grains were isolated from Russian kefir drink which was obtained from a local market [28]. The kefir grains were gently separated from the drink by a strainer under aseptic conditions. Subsequently, the grains were activated in laboratory using pasteurized cow milk at 30 °C through anaerobic fermentation for 48 h. The produced kefir grains were harvested by centrifugation at 5000 rpm for 10 min.

### 2.2. Isolation of LAB Strains

Lactic acid bacteria were isolated from kefir grains cultured at the Laboratory of Microbiology, Biotechnology and Food Hygiene of the Department of Agricultural Development of Democritus University of Thrace. Kefir grains (25 g) were aseptically weighted into filtered stomacher bags and homogenized with 225 mL (0.1% *w/v*) peptone water. Samples were then serially diluted and 1 mL of dilution was incorporated into de Man, Rogosa, and Sharp (MRS) agar (Merck, Darmstadt, Germany). MRS plates were incubated at 37 °C for 48–72 h. All isolates were further purified by streak plating and preliminarily identified based on their morphological and staining characteristics (Gram-positive bacilli). In addition, negative catalase reaction (3% *v/v* H_2_O_2_) was applied. All lactobacilli were stored at −80 °C in MRS broth (Merck) tubes supplemented with 30% sterile glycerol.

### 2.3. Bacterial Culture Conditions

All *L. paracasei* strains were obtained from DSMZ (Braunschweig, Germany). *L. casei* ATCC 393 and *L. plantarum* ATCC 14917 were obtained from ATCC (LGC Standards, Middlesex, UK). *L. paracasei* 20008, *L. paracasei* 20207 and *L. paracasei* 46331 strains were grown at 30 °C while ATCC strains were grown anaerobically at 37 °C. In all cases MRS broth (Merck) was used as culture media.

### 2.4. In Vitro Tests Simulating the Human GI Tract

#### 2.4.1. Resistance to Low pH

Resistance to low pH was evaluated in terms of viable colony counts and enumerated on MRS agar plates after incubation at 37 °C for 0 and 2 h [21]. The adjustments of pH values (2, 3, and 4) were made using 5 M HCl. The experiment was done in triplicates and the results are presented as average values plus standard deviations.

#### 2.4.2. Resistance to Pepsin and Pancreatin

Resistance of the lactobacilli to pepsin and pancreatin was tested as described previously [21]. In particular, bacterial cells from overnight cultures were harvested through centrifugation (10,000× *g*, 5 min, 4 °C), washed twice with PBS and then resuspended in PBS solution pH 2.0 containing pepsin (3 mg/mL; Sigma, St. Louis, MO, USA). In the same manner, bacterial cells were resuspended in PBS solution pH 8.0 containing pancreatin USP (1 mg/mL; Sigma). Resistance was determined in terms of viable colony counts on MRS agar plates and enumerated after incubation at 37 °C for 0 and 3 h with pepsin, and 0 and 4 h with pancreatin. The experiment was done in triplicates and the results are presented as average values plus standard deviations.

#### 2.4.3. Tolerance to Bile Salts

Tolerance to bile salts was determined as reported previously by Plessas et al. (2017) [21]. In brief, bacterial cells were cultured overnight for 18 h and harvested by certification (10,000× *g*, 5 min, 4 °C). The harvested cells were washed twice with PBS buffer (pH 7.2), before being resuspended in PBS solution (pH 8.0), containing 0.5% (*w/v*) bile salts. Resistance was assessed in terms of viable colony counts and enumerated after incubation for 0 and 4 h at 37 °C. The conditions were selected targeting simulation of the time spent by food in the small intestine [29]. The experiment was done in triplicates and the results are presented as average values plus standard deviations.

#### 2.4.4. Antibiotic Susceptibility Testing

The LAB strains were examined for their susceptibility against ten common antibiotics (amoxycillin, amoxycillin/clavulanic acid, ampicillin, clindamycin, erythromycin, gentamicin, metronidazole, tetracycline, tigecycline, and vancomycin) with concentration from 0.015 to 256 μg/mL, as described previously [21]. MICs (μg/mL) were determined by gradient diffusion using M.I.C. Three replicates per strain were conducted. *L. plantarum* ATCC 14917 was used as a reference strain.

### 2.5. DNA Extraction from Pure Cultures

Genomic DNA was extracted from pure bacterial cultures using a DNeasy Tissue kit (Qiagen, Hilden, Germany). The amount and purity of the extracted DNA was determined spectrophotometrically.

### 2.6. PCR Amplification

PCR reactions were carried out as reported by Plessas, et al. (2017) [21]. The primers P1 and P2 were described by Klijn, et al. [30]. The reactions were analyzed by electrophoresis on 1% *w/v* agarose gels stained with 0.5 μg/mL ethidium bromide, visualized under UV illumination and photographed with a digital camera (GelDoc EQ system, Biorad, Segrate, Italy). The PCR products were purified using a PCR extraction kit (Macherey-Nagel, Düren, Germany). Following sequencing of the PCR products (VBC-Biotech, Vienna, Austria), BLAST analysis was performed as described before [21].

### 2.7. Species-Specific Multiplex PCR

Species-specific multiplex PCR was performed as reported previously by Plessas, et al. (2017) [21]. Primers PAR, CAS, RHA, and 50 CPR were described by Ventura, et al. [31]. The reactions were analyzed by electrophoresis on 1% *w/v* agarose gels stained with 0.5 μg/mL ethidium bromide, visualized under UV illumination and photographed with a digital camera (GelDoc EQ system, Bio-Rad).

### 2.8. Application of *L. paracasei* SP3 for Feta-Type Cheese Production

Feta-type cheese with *L. paracasei* SP3 cells was produced using pasteurized and standardized ovine milk (70%) and goat’s milk (30%) (paracasein-to-fat ratio: 0.7, pH 6.7 and lactose: 4.5%) from a local dairy factory (Chelmos S.A., Achaia, Greece). *L. paracasei* SP3 (3.2 g/L of milk, resulting in approximately 10 log cfu/L of milk) was added to milk heated at 37 °C. After 30 min, commercial rennet (0.01%) was added and the mixture was left undisturbed for 2 h for curd formation. The curd was cut in squares (diameter 1 cm) and left undisturbed for 10 min. Then samples were placed into sterile cubic cheese molds and conducted periodic stirring for 12 h at room temperature (17–20 °C) to facilitate whey drainage [32]. Subsequently, the cheese was removed from the molds, left undisturbed for 10 min and placed into brine (12% salt) at room temperature (17–20 °C) until pH dropped to 4.6. Cheese samples were monitored by a digital pH meter (HI 99161, Hanna Inc., Athens, Greece) and when the pH dropped after approx. 30–45 days the brine was replaced (6% salt) and cheese samples were placed at 4 °C for further maturation [32]. Feta-type cheese samples produced by *L. paracasei* SP3 (FSP3) were compared with cheese samples prepared by commercial rennet enzyme (FC) (Chelmos S.A.). All treatments were carried out in triplicate and the mean values are presented. Ripening of cheeses was studied during maturation (60 days) and storage (4 °C) for up to 70 consecutive days. Samples from each treatment were collected at various intervals (0, 1, 5, 14, 30, 45, and 70 d) and were subjected to physicochemical and microbiological analysis.

### 2.9. Physicochemical Analysis of Feta-Type Cheese

Cheese pH was measured using a digital pH meter (HI 99161, Hanna Inc.). Moisture content, ethanol content, and total acidity (expressed as lactic acid content) of cheese samples were determined according to AOAC International (2010) [33]. Total nitrogen in DM was determined using the Kjeldahl procedure [13]. Sugars and organic acids were analyzed by high performance liquid chromatography (HPLC). Cheese samples (20 g each) were treated with warm water (40 °C) to produce a total volume of 210 mL. Then samples were filtered and submitted for organic acid and sugar analysis. For the determination of organic acids, the filtered samples were treated with 40% trichloroacetic acid (TCA) for protein precipitation. In briefly, 9 mL of the suspensions of the filtrate were treated with 1 mL of TCA and left to stand for 24 h at 4 °C [10]. The treated samples were centrifuged at 5000 rpm for 30 min at 4 °C. All samples were filtered through 0.2 μm nylon filters prior analysis. Lactic acid was analyzed on a Jasco LC-2000 Series HPLC system (Jasco Inc., Tokyo, Japan) equipped with a size-exclusion organic acid analysis column (Aminex HPX-87H, 300 × 7.8 mm i.d., 9 μm particle size, Bio-Rad) fitted in a CO-2060 Plus column oven, a PU-2089 pump, a AS 2050 Plus autosampler and a MD-2018 Photodiode array detector operated at 210 nm. Isocratic separation at 50 °C was performed with 0.008 N H_2_SO_4_ as mobile phase at a flow rate of 0.6 mL/min. The detector signals were recorded and analyzed by ChromNav software. Standard solutions of acids (Sigma) were used for quantitative analysis and were prepared at various concentrations in pure water. Lactose, glucose and galactose were determined on a Shimadzu chromatograph (Kyoto, Japan) with a Nucleogel Ion 300 OA column, a LC-9A pump, a CTO-10A oven at 40 °C and a RID-6A refractive index detector. As a mobile phase used was used 0.008 N H_2_SO_4_ with a flow rate of 0.5 mL/min. 1-propanol (0.1% *v/v*) was used as an internal standard [32]. The sample dilution was 1% *v/v* and was filtered with disposable 0.20 nm cellulose acetate filters (Chromafil, Macherey-Nagel) prior injection. Subsequently, 60 μL of the filtrates were injected directly to the column [32]. Sugar concentrations were calculated using standard curves.

### 2.10. Microbiological Analysis of Feta-Type Cheese

Representative duplicate 10 g portions were retrieved from each cheese sample during various time intervals and blended with 90 mL of sterilized 2% *w/v tri*-sodium citrate solution. The suspension was then submitted to 10 decimal serial dilutions with ¼ strength Ringer’s solution. Viable counts of total aerobic bacterial counts, Lactococci, Lactobacilli, yeasts and fungi, and coliforms were determined in triplicate by pour plating of appropriate dilutions (0.1 mL or 1 mL) on the selective media for each species according to instructions of the manufacturer [26]. Specifically, viable counts of total aerobic bacterial counts were enumerated on plate count agar (PCA) (Merck) by suspended 0.1 mL of the sample after incubation at 30 °C for 72 h. Lactococci were enumerated on M-17 agar (Merck) by suspended 1 mL of the sample after incubation 30 °C for 48–72 h. Lactobacilli were enumerated on MRS agar (Merck) by suspended 1 mL of the sample after incubation at 37 °C for 48 h. Yeasts and fungi were determined by suspended 0.1 mL of the sample after incubation on Potato Dextrose agar (PDA) (Merck) at 30 °C for 72 h. Finally, coliforms were enumerated on Violet Red Bile agar (LabM, Heywood, U.K.) by suspended 1 mL of the sample after anaerobic incubation at 30 °C for 24 h. All cell counts were expressed as log of mean colony forming units (CFU).

### 2.11. Preliminary Sensory Evaluation

Sensory evaluation of Feta-type cheese was conducted to evaluate the influence of *L. paracasei* SP3 on cheese preliminary sensory characteristics. Sensory evaluation was carried out by 10 laboratory members, priory trained, using locally-approved protocols [12]. Cheese samples were evaluated during the 70th day of storage regarding color, cheese odor, saltiness, acidity, bitterness, sweetness, chewiness, and overall acceptability. Sensory evaluation was carried out by 10 laboratory members between 20 and 55 years of age which were frequent consumers of feta cheese (>once a week). Cheese samples (FSP3 and FC) were collected from the 70th storage day (4 °C), placed into equivalent amounts (5 × 5 cm), and served at room temperature (18–22 °C). Sensory analysis was carried out in panel booths while the evaluators were unaware of the samples they tasted [12]. The results are based on a 0–10 preference scale and are presented as a star chart of the product’s attributes.

### 2.12. Statistical Analysis

Cheese production was carried out in triplicate and results of the physicochemical and sensory evaluation tests are presented as the mean ± standard deviation of their corresponding values. Microbial counts are expressed as mean Log_10_ colony forming units per gram of cheese. Results among the various treatments between the 10 strains and the reference were compared by using either the *t*-student procedure (for two datasets) or the analysis of variance (ANOVA) with the least significant difference (LSD) for multiple range testing both at a 95% confidence level. All statistical analyses were performed with IBM^®^ SPSS^®^ Statistics v.20 (IBM Corp. Armonk, NY, USA).

## 3. Results and Discussion

### 3.1. Isolation of LAB Strains and Screening for Probiotic Potential

An important step towards the selection of potential probiotic candidates is to investigate the strain behavior under conditions which mimic the gastrointestinal tract of the consumer. When a potential probiotic microorganism is consumed its viability can mostly be infected by the low pH (1.5~3.0) of stomach and the bile contained in the upper intestine [34]. Likewise, 52 strains were isolated from kefir grains in the present study. Most of them (73%) were categorized to the genus of *Lactobacillus*. Therefore, 38 LAB strains were screened in a series of established in vitro tests for probiotic potential such as: (i) resistance to low pH, (ii) resistance to pepsin and pancreatin, and (iii) tolerance to bile salts. *Lactobacillus plantarum* ssp. *plantarum* ATCC 14971 was selected and employed in the above in vitro tests as a reference probiotic strain [35,36]. The outcome showed that 10 of these strains displayed probiotic potential (Table 1). Regarding resistance to low pH only SP3 presented satisfactory levels of viability (7.1~8.5 log cfu/mL) in the pH values between 2.0 and 4.0, which is considered as significant prerequisite for probiotic delivery (Table 1). Digestion process significantly influences the amount of viable probiotic microorganisms able to reach the intestine and colonize [2]. The pH of the gastric juice is considered among the main factors affecting their survival upon passage through the stomach. Interestingly this newly isolated strain presented higher viability levels in pH 4.0 compared to the reference strain *L. plantarum* ATCC 14971 (*t* = 4.03, *p* = 0,015). Concerning resistance to pepsin and pancreatin and tolerance to bile salts, the strain SP3 exhibited the best performance among the ten strains and their achieved viabilities were similar and even better to the respective values of the reference strain (Table 1). These results indicate that the novel SP3 strain is likely to survive thought the gastrointestinal tract and can be further investigated for its potential as a probiotic culture.

### 3.2. Antibiotic Susceptibility

The outcome regarding MIC (μg/mL) values of the most promising for probiotic action, *Lactobacillus* strains as well the MIC of *L. plantarum* ATCC 14917 against ten common synthesized antibiotics is presented at Table 2. All lactobacilli were resistant to vancomycin (MIC > 256 μg/mL) and tetracycline (MIC > 4 μg/mL). Additionally, seven out of the ten strains tested were resistant to clindamycin (MIC > 1 μg/mL) and erythromycin (MIC > 1 μg/mL). Minimum inhibitory concentration ranged from 1.58 to 4.41 μg/mL for amoxicillin, 0.18–2.67 μg/mL for amoxicillin-clavulanic acid, 0.38–2.08 μg/mL for ampicillin, 5.18–9.15 μg/mL for gentamycin, 77.9–200.1 μg/mL for metronidazole, and 0.35–0.64 μg/mL for tigecycline. Among the tested strains, SP3 strain showed the lower MIC values for ampicillin, clindamycin, tetracycline and tigecycline. An inherent resistant to vancomycin and metronidazole similar to our results has been reported in the past [21,37]. Resistance of Lactobacilli, Pediococci, and *Leuconostoc* spp. to vancomycin is due to a natural property of the above species arise by the presence of d-alanine:d-alanine ligase related enzymes [38]. In the same way, a natural or ‘intrinsic’ resistance of lactobacilli to metronidazole (nucleic acid synthesis inhibitor) has also been reported [39]. Additionally, in our study a shared resistance to tetracycline was also observed by all *Lactobacillus* strains studied here, which is in accordance with other researchers [40,41].

### 3.3. Molecular Characterization of Lactobacillus Strain SP3

**Figure 1:**
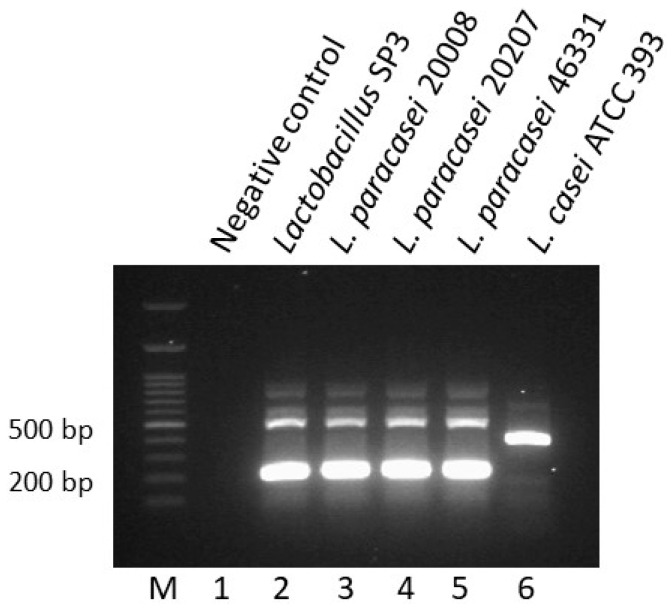
Species-specific multiplex PCR for *Lactobacillus paracasei* SP3.

The in vitro tests demonstrated that *Lactobacillus* strain SP3 possesses the best probiotic potential of all strains tested. To characterize strain SP3 at species-level, a variable region of the 16S rRNA gene was amplified and the sequence of the PCR product was introduced in GenBank. The BLAST analysis revealed that SP3 showed 99% similarity to different *L. paracasei* and *L. casei* species (Table S1). To further characterize strain SP3, a species-specific multiplex PCR with primers PAR, CAS, RHA, and CPR was performed [16,25]. The PCR product of strain SP3 shows the unique pattern of the PCR products of the three *L. paracasei* reference strains (Figure 1). Therefore, the strain SP3 is affiliated to this *Lactobacillus* species.

### 3.4. L. paracasei SP3 as Starter Culture for the Production of Feta-Type Cheese

Traditionally, Feta cheese used to be produced from raw milk in small family premises. Fresh raw milk was coagulated by rennet enzyme from abomasa of lambs and kids and coagulation ranged between 50 min to 1 h [42]. Producers occasionally used to heat raw milk and add traditional yogurt culture as a starter. On the other hand, Feta which is a semi-soft, white brined, traditional Greek cheese is produced in an industrial scale from pasteurized and standardized milk with the addition of various commercial starters [43]. Targeting to evaluate its technological potential, the strain *L. paracasei* SP3 was applied as starter culture for Feta-type cheese production.

The physicochemical parameters of Feta-type cheeses produced with the use of *paracasei* SP3 as starter compared with Feta cheese produced with rennin enzyme are presented in Table 3. All parameters ranged in levels usually observed in commercial Feta cheese [13,32,42]. In is noteworthy, that *L. paracasei* SP3 and the ripening time seems to affect all parameters studied. In addition, a strong interaction was observed by the use of *L. paracasei* SP3 as starter affecting sugars, pH, and acidity in comparison with cheese produced by rennin enzyme. Moreover, total acidity expressed as lactic acid content was observed much higher during maturation and storage in cheese samples produced with *L. paracasei* SP3 in addition to pH values with were detected in higher values compared with cheese samples produced by rennin enzyme (no starter culture). A fast pH decrease along with enhanced lactic acid production is very important in white brine cheese production as low pH inhibits the growth of foodborne pathogens and various spoilage microorganisms. The high Lactobacilli rates which were reported during the 1st ripening period of Feta-type cheese (Table 4), may be considered responsible for the enhanced total acidity and the fast pH decrease; results which have also been highlighted by previous studies regarding Feta-type cheese production [26,32]. In general, post acidification was observed during storage (4 °C) of Feta-type cheese produced with *L. paracasei* starter which is in accordance with previous studies of white brined cheeses produced with the incorporation of lactic acid bacteria [26,32,44].

Feta-type cheeses of the present study fulfilled the Greek Codex Alimentarius requirements of high-quality white-brined cheeses regarding moisture content that should be ≤ 56%. Specifically, moisture content ranged between 60 and 50% (*wt/wt*) in the produced Feta-type cheeses during maturation and storage for a total of 70 days. Additionally, moisture content was affected by the addition of *L. paracasei* SP3 starter culture and the ripening process. Similar results have also been found in various Feta-type and white brine cheeses [13,45,46]. In general, it can be assumed that low pH values resulted in whey removal from cheese into the brine while the high salt concentration of the brine was incorporated into cheese products resulting in lower moisture content of all cheese samples during manufacture and storage. This procedure leads to a firm well-conducted brined cheese adversely affecting cheeses’ sensorial quality [47]. Finally, total nitrogen in DM, determined after 70 days, was observed higher in cheese samples produced with *L. paracasei* starter culture compared with cheese with no starter culture. This result is in agreement with previous studies reporting that lactic acid bacteria used in cheese production can enhance nitrogen concentration thought proteolysis of the final product as they remain viable during ripening and storage [13,48].

### 3.5. Microbiological Analysis of Feta-Type Cheese

The association of the microbial groups examined during the 1st (room temperature) and 2nd (4 °C) ripening period as well as during storage (4 °C) of Feta-type cheese samples is presented in Table 4. In general, there was detected an increase in all microbial counts during the 1st ripening period (maturation at room temperature) in all cheese samples. It is noteworthy that lactobacilli count showed high increasing rates during 70 days of ripening and storage while they were found to be above 6 log CFU/g in commercial cheese and approx. 7.9 log CFU/g in potential probiotic Feta-type cheese during the 1st day of manufacture. In parallel, as lactobacilli microbial rates were found to be enhanced during ripening and storage period, other microbial groups like lactococci, coliforms, yeast and fungi were found to be decreased especially in cheese samples manufacture with *L. paracasei* SP3 starter culture. During the 2nd ripening period and during storage (4 °C), a shift in microorganism population was observed. Specifically, as total aerobic counts decreased in all trials, coliforms, and yeasts/fungi populations significantly decreased only in the samples inoculated with the starter potential probiotic strain. This result is in accordance with previous studies indicating that lactic acid bacteria dominate against other microorganisms when they are active in the curd during cheese manufacture [26,49].

Lactococci are considered indigenous milk microflora as they dominate raw milk within the first 24 h of production. Lactococci counts were significantly affected by time of ripening and storage as well as by the starter culture. Specifically, during the 2nd storage period and refrigerated storage period lactococci counts ranged between 7.32 and 6.58 log cfu/g in commercial cheese samples while their counts were detected significantly lower in cheese samples produced by *L. paracasei* SP3 starter culture (6.67~5.02 log cfu/g).

There was detected a drastic decrease in coliform, yeast and fungi numbers during ripening and storage period especially in Feta-type cheeses produced with the novel potential probiotic stain. This result is in accordance with previous studies reporting depression of spoilage and pathogenic bacteria by probiotic lactic acid bacteria [50,51]. Yeasts and fungi are not part of the starter cultures used in white brine cheesemaking as is Feta cheese [27]. However, relatively high numbers of yeast and fungi can be detected in many soft and semi-hard ripened cheeses as they naturally occur in the environment and can easily colonize in milk products. The presence of yeasts in cheese products is related to ethanol production so we can assume that the small amount of ethanol detected in Feta-type cheese products (Table 3) might be derived from yeast accumulation. Although yeasts and fungi were present in all cheese samples during maturation and storage, their numbers were detected significantly higher during storage period in cheese produced without no starter culture. As a result, it can be assumed that the development of conditions favors their growth while the action of *L. paracasei* SP3 culture could inhibit their growth during cheese maturation and storage. Recent scientific evidence has drawn to the conclusion that LAB exhibit antimicrobial effects against spoilage and pathogenic microorganisms by the production of acids, antifungal peptides, and bacteriocins [26]. Several studies have reported the inhibitory effect of *L. paracasei* strains against various pathogenic and spoilage microorganisms, including yeasts and fungi [13,52,53,54]. To conclude, among many studies is reported the antimicrobial activity of LAB against various pathogenic or possible spoilage microorganisms, indicating that LAB could be used as a biocontrol agent in food production [12,51,55,56].

### 3.6. Preliminary Sensory Evaluation

**Figure 2:**
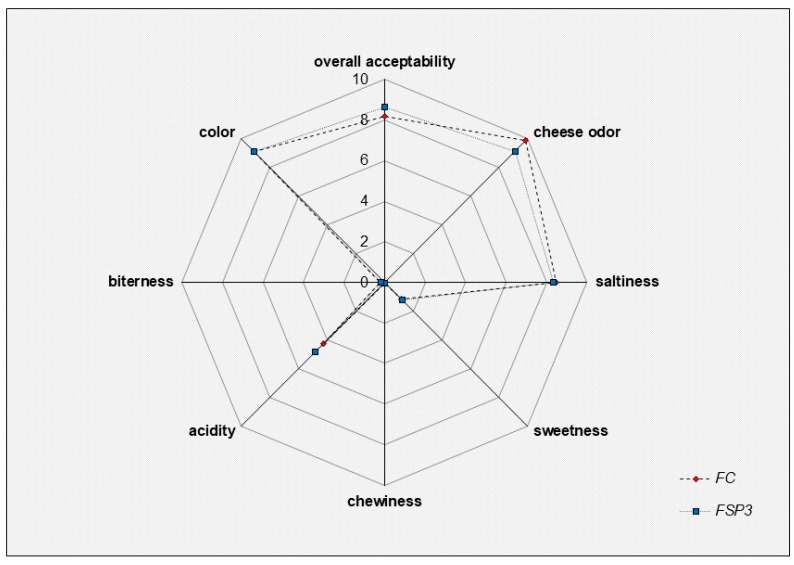
Sensory evaluation of produced Feta-type chesses presented as a spider chart of products attributes. *FC*: (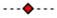) Feta-type cheese produced with rennin enzyme and used as a control sample. *FSP3*: (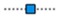) Feta-type cheese produced with *L. paracasei* SP3 as starter culture.

The results of sensory evaluation of Feta-type cheese samples are presented in Figure 2. Assessors evaluated the produced Feta-type cheese regarding color, cheese odor, saltiness, acidity, bitterness, sweetness, chewiness, and overall acceptability. In general, all cheese samples were characterized by high rates of acceptance. In most cases, no significant differences were observed between FC and FSP3 cheese samples. FSP3 cheese retrieved higher rates in acidity and overall acceptance compared to commercial Feta cheese produced with no starter culture (FC). In addition, Feta-type cheese with *L. paracasei* SP3 were characterized by assessors as firm and more consistent compared to commercial cheese samples. These findings indicated the high industrialization potential of the proposed technology since sensory evaluation showed consumers preference while an overall improvement in cheese quality was evident. The use of Lactobacilli strains with probiotic potential has also been reported by other studies to provide desirable and robust technological properties [44] in cheese production, thus, isolated lactobacilli, as is *L. paracasei* SP3, are most likely to be used in cheese production targeting the development of functional cheese products with optimum technological characteristics.

## 4. Conclusions

In the frame of the present study, a novel *Lactobacillus paracasei* SP3 isolated from kefir grains exhibited desirable probiotic properties and was successfully employed as starter culture for feta-type cheese production. The produced Feta-type cheese had higher acidity, lower pH, reduced counts of coliforms, yeasts and fungi, and improved quality characteristics compared with cheese produced with no starter culture. Therefore, *Lactobacillus paracasei* SP3 displayed acceptable technological properties for novel probiotic food production. We are currently investigating the beneficial health effects of *Lactobacillus paracasei* SP3 employing in vitro and in vivo models to establish its probiotic character.

## Figures and Tables

**Table 1:** Assessment of viability of the isolated strains after exposure to low pH, bile salts, pepsin and pancreatin. In all tests, the probiotic *L. plantarum* 14917 served as a reference strain.

	Final Counts (log cfu/mL)											
	Isolated *Lactobacillus* Strains											
	Time (h)	SP3	SP16	SP18	SP10	SP21	SP22	SP27	SP30	SP33	SP36	*L. plantarum* ATCC 14971
**Resistance to low pH**	0	8.9 ± 0.10	9.0 ± 0.23	8.8 ± 0.13 *	8.6 ± 0.20	8.9 ± 0.17	8.3 ± 0.24 *	8.1 ± 0.24 *	8.2 ± 0.15	8.5 ± 0.25 *	8.4 ± 0.18 *	9.1 ± 0.21
**pH = 2**	2	7.1 ± 0.13 *	3.2 ± 0.12 *	2.5 ± 0.41 *	1.7 ± 0.19 *	1.3 ± 0.23 *	1.8 ± 0.11 *	0	0	0	0	7.9 ± 0.15
**pH = 3**	2	7.6 ± 0.11	6.9 ± 0.12 *	7.3 ± 0.26 *	6.5 ± 0.07 *	6.9 ± 0.08 *	8.0 ± 0.13	8.6 ± 0.23 *	7.8 ± 0.11	6.9 ± 0.23 *	6.8 ± 0.15 *	7.8 ± 0.05
**pH = 4**	2	8.5 ± 0.14 *	8.3 ± 0.19	7.7 ± 0.12 *	7.9 ± 0.11	7.8 ± 0.11 *	8.5 ± 0.21 *	7.6 ± 0.11 *	7.2 ± 0.17 *	7.3 ± 0.08 *	7.2 ± 0.09 *	8.1 ± 0.10
**Pepsin**	0	7.4 ± 0.15	7.6 ± 0.23	7.1 ± 0.29	7.2 ± 0.13	7.3 ± 0.15	7.2 ± 0.29	7.1 ± 0.18	7.3 ± 0.21	7.2 ± 0.13	7.5 ± 0.11	7.3 ± 0.05
	3	6.6 ± 0.14	6.6 ± 0.06	4.7 ± 0.11 *	5.1 ± 0.18 *	4.9 ± 0.15 *	5.2 ± 0.25 *	5.6 ± 0.22 *	3.3 ± 0.19 *	3.8 ± 0.23 *	4.5 ± 0.23 *	6.7 ± 0.15
**Pancreatin**	0	8.8 ± 0.11 *	8.3 ± 0.17	8.0 ± 0.26	8.4 ± 0.18	8.0 ± 0.17	8.1 ± 0.14	8.2 ± 0.21	8.3 ± 0.13	8.2 ± 0.15	8.5 ± 0.17 *	8.2 ± 0.10
	4	7.3 ± 0.05	5.1 ± 0.19	4.2 ± 0.07	5.6 ± 0.15	5.3 ± 0.15	4.5 ± 0.15	5.0 ± 0.19	5.4 ± 0.28	5.2 ± 0.11	5.2 ± 0.11	7.5 ± 0.10
**Bile salts**	0	8.8 ± 0.08	8.6 ± 0.27	8.6 ± 0.12	8.5 ± 0.10	8.5 ± 0.11	8.7 ± 0.29	8.5 ± 0.13	8.5 ± 0.21	8.9 ± 0.11	8.4 ± 0.19	8.7 ± 0.20
	4	8.5 ± 0.05 *	7.0 ± 0.31 *	6.4 ± 0.27 *	7.1 ± 0.11 *	7.2 ± 0.19 *	6.4 ± 0.27 *	6.3 ± 0.17 *	7.1 ± 0.27 *	6.2 ± 0.09 *	5.5 ± 0.21 *	8.0 ± 0.15

* Denotes a statistically significant difference in viability between the corresponding strain and *L. plantarum* ATCC 14971 (reference) for each treatment.

**Table 2:** MIC (μg/mL) of antibiotics for specific *Lactobacillus* strains as determined by gradient diffusion using M.I.C. Evaluator^®^ strips. The probiotic *L. plantarum* 14917 served as a reference strain.

Agent	SP3	SP16	SP18	SP10	SP21	SP22	SP27	SP30	SP33	SP36	*L. plantarum* ATCC 14917	Cut-Off ^a^
	(MIC μg/mL)											
**Amoxycillin**	2.28 ± 0.27	1.58 ± 0.11 *	3.7 ± 0.11	3.84 ± 0.31 *	4.41 ± 0.79 *	2.76 ± 0.91	3.10 ± 0.17	4.15 ± 0.23 *	3.75 ± 0.29 *	3.84 ± 0.59 *	2.86 ± 0.78	n.r. ^b,c^
**Amoxycillin + Clavulanic acid**	1.57 ± 0.31	1.14 ± 0.15 *	1.76 ± 0.15 *	1.14 ± 0.13 *	0.49 ± 0.05 *	1.44 ± 0.19 *	1.95 ± 0.11 *	1.25 ± 0.51 *	0.84 ± 0.11 *	1.24 ± 0.29 *	2.67 ± 0.15	n.r. ^b,c^
**Ampicillin**	0.38 ± 0.07	0.99 ± 0.05 *	1.26 ± 0.09 *	2.08 ± 0.19 *	1.76 ± 0.21 *	1.76 ± 0.09 *	1.29 ± 0.23 *	1.47 ± 0.09 *	0.76 ± 0.08 *	0.76 ± 0.31 *	0.58 ± 0.33	4 ^b^
**Clindamycin**	0.73 ± 0.05	1.14 ± 0.26 *	1.54 ± 0.20 *	0.96 ± 0.08	2.29 ± 0.21 *	1.33 ± 0.21 *	1.12 ± 0.10 *	1.51 ± 0.15 *	1.95 ± 0.21 *	0.97 ± 0.13 *	0.67 ± 0.20	1 ^b^
**Erythromycin**	0.34 ± 0.11 *	0.43 ± 0.09 *	1.08 ± 0.11	1.79 ± 0.15 *	1.89 ± 0.09 *	1.81 ± 0.15 *	0.35 ± 0.05 *	1.23 ± 0.19	1.74 ± 0.19 *	1.41 ± 0.10	1.00 ± 0.87	1 ^b^
**Gentamycin**	5.18 ± 0.12 *	6.57 ± 1.15 *	5.73 ± 0.31 *	8.41 ± 0.71 *	7.29 ± 0.31 *	9.15 ± 0.47 *	8.71 ± 1.11 *	5.21 ± 0.79 *	6.43 ± 0.31 *	6.08 ± 1.71 *	3.33 ± 1.15 *	32 ^b^
**Metronidazole**	103.4 ± 22.7 *	144.1 ± 21.9 *	175.2 ± 30.4 *	148.1 ± 3.08 *	77.9 ± 10.23 *	104.4 ± 18.3 *	200.1 ± 21.8 *	153.1 ± 21.39 *	148.3 ± 20.15 *	138.1 ± 19.2 *	>256	n.r. ^b,c^
**Tetracyclinne**	4.13 ± 0.10 *	5.29 ± 0.39 *	9.55 ± 0.81 *	5.36 ± 0.71 *	6.23 ± 0.58 *	4.48 ± 0.93 *	5.12 ± 0.76 *	6.53 ± 0.87 *	10.89 ± 0.95 *	14.08 ±1.39	13.3 ± 4.62	4 ^b^
**Tigecycline**	0.35 ± 0.05	0.47 ± 0.08	0.41 ± 0.15	0.49 ± 0.08	0.64 ± 0.07 *	0.59 ± 0.11 *	0.62 ± 0.13 *	0.59 ± 0.11 *	0.61 ± 0.08 *	0.44 ± 0.05	0.33 ± 0.14	n.r. ^b,c^
**Vancomycin**	>256	>256	>256	>256	>256	>256	>256	>256	>256	>256	>256	n.r. ^b,c^

* Denotes a statistically significant difference in MIC values between the corresponding and the reference strain (*L. plantarum* ATCC 14971). ^a^ Breakpoints are referred to L. casei/paracasei strains. EFSA breakpoints for other types of LABs are slightly different. ^b^ Strains with MIC higher than the breakpoints are considered as resistant according to EFSA. ^c^ not required.

**Table 3:** Effect of *Lactobacillus paracasei* SP3 on physicochemical characteristics and sensory attributes of Feta-type cheese.

Cheese	Ripening Period (Days)	Lactose (g/100 g of Cheese)	Glucose (g/100 g of Cheese)	Galactose (g/100 g of Cheese)	Ethanol (g/100 g of Cheese)	pH	Acidity (g of Lactic Acid/100 g of Cheese)	Moisture (%, *wt/wt*)	Total N in DM (%)
No starter culture	0	3.85 ± 0.10	0.19 ± 0.03	0.26± 0.05	0.03 ± 0.01	6.50 ± 0.10	0.13 ± 0.02	60.0 ± 1.5	
	1	3.78 ± 0.15	0.06 ± 0.02	0.15± 0.02	0.04 ± 0.01	6.48 ± 0.05	0.14 ± 0.02	51.5 ± 2.0	
	5	3.50 ± 0.05	Tr1	Tr	0.12 ± 0.01	6.44 ± 0.10	0.18 ± 0.02	45.1 ± 2.5	
	14	2.24 ± 0.15	Tr	Tr	0.15 ± 0.01	5.75 ± 0.10	0.53 ± 0.03	56.0 ± 2.5	
	30	1.84 ± 0.10	Tr	Tr	0.17 ± 0.02	5.60 ± 0.10	0.39 ± 0.01	54.5 ± 1.0	
	45	1.20 ± 0.05	Tr	Tr	0.11 ± 0.01	5.62 ± 0.10	0.30 ± 0.02	54.9 ± 1.0	
	70	0.78 ± 0.05	Tr	Tr	0.08 ± 0.01	5.48 ± 0.10	0.20 ± 0.01	54.1 ± 1.5	4.89 ± 0.10
*L. paracasei* SP3	0	1.95 ± 0.05 *	0.32 ± 0.04	0.27± 0.04	0.03 ± 0.01	6.18 ± 0.10	0.21 ± 0.02 *	60.5 ± 2.3	
	1	1.58 ± 0.05 *	0.12 ± 0.03	0.19± 0.03	0.03 ± 0.01	5.32 ± 0.10 *	0.42 ± 0.05 *	50.1 ± 2.1	
	5	1.32 ± 0.07 *	0.06 ± 0.02 *	0.06± 0.02 *	0.04 ± 0.01 *	4.95 ± 0.05 *	0.62 ± 0.05 *	55.0 ± 1.1 *	
	14	0.65 ± 0.07 *	Tr	Tr	0.08 ± 0.01 *	4.78 ± 0.10 *	0.80 ± 0.07 *	50.1 ± 1.5 *	
	30	0.32 ± 0.08 *	Tr	Tr	0.10 ± 0.01 *	4.80 ± 0.10 *	0.91 ± 0.09 *	51.9 ± 1.0 *	
	45	0.15 ± 0.10 *	Tr	Tr	0.09 ± 0.02 *	4.72 ± 0.10 *	0.89 ± 0.05 *	50.5 ± 3.7 *	
	70	Tr	Tr	Tr	0.04 ± 0.01 *	4.62 ± 0.05 *	0.90 ± 0.05 *	50.5 ± 1.8 *	6.22 ± 0.09 *

* Denotes a statistical significant difference between cheese samples without starter culture and with SP3 as a starter culture for the corresponding ripening period. Tr = Traces.

**Table 4:** Effect of *Lactobacillus paracasei* SP3 on major microbial groups of Feta-type cheese.

Cheese Type	Ripening and Storage Period (d)	Total Aerobic Count (log cfu/g)	Lactococci (log cfu/g)	Lactobacilli (log cfu/g)	Yeasts & Fungi (log cfu/g)	Coliforms (log cfu/g)
**No starter culture**	0	5.55 ± 0.15	5.78 ± 0.20	6.17 ± 0.35	5.05 ± 0.20	4.65 ± 0.15
	1	6.25 ± 0.25	6.95 ± 0.25	6.50 ± 0.19	6.01 ± 0.18	5.21 ± 0.20
	4	8.95 ± 0.35	6.54 ± 0.18	6.95 ± 0.24	7.15 ± 0.30	5.12 ± 0.35
	15	9.55 ± 0.30	7.32 ± 0.21	7.75 ± 0.29	7.21 ± 0.28	4.58 ± 0.30
	30	8.75 ± 0.30	7.11 ± 0.32	7.21 ± 0.32	6.55 ± 0.25	5.10 ± 0.29
	45	8.20 ± 0.48	6.99 ± 0.28	7.11 ± 0.28	6.12 ± 0.31	4.69 ± 0.25
	70	7.95 ± 0.34	6.58 ± 0.24	7.05 ± 0.30	5.25 ± 0.32	4.37 ± 0.20
***Lactobacillus paracasei* SP3**	0	6.12 ± 0.39	5.82 ± 0.34	7.93 ± 0.28 *	5.10 ± 0.19	4.90 ± 0.19
	1	6.85 ± 0.33	6.21 ± 0.37 *	8.12 ± 0.35 *	6.10 ± 0.24	5.12 ± 0.31
	4	8.93 ± 0.29	6.88 ± 0.26	8.75 ± 0.28 *	6.65 ± 0.26	4.42 ± 0.30
	15	9.58 ± 0.38	6.67 ± 0.33 *	9.02 ± 0.22 *	5.85 ± 0.24 *	4.05 ± 0.25
	30	9.05 ± 0.34	5.12 ± 0.34 *	8.86 ± 0.38 *	4.95 ± 0.24 *	3.56 ± 0.41 *
	45	8.59 ± 0.36	5.12 ± 0.29 *	8.43 ± 0.24 *	3.77 ± 0.35 *	3.07 ± 0.35 *
	70	8.42 ± 0.32	5.02 ± 0.35 *	8.18 ± 0.32 *	3.19 ± 0.39 *	2.15 ± 0.34 *

* Denotes a statistically significant difference in microbial counts between cheese samples without starter culture and with *Lactobacillus paracasei* SP3 as a starter culture for the corresponding ripening period.

## Supplementary Materials

The following are available online at http://www.mdpi.com/2076-2607/6/4/121/s1, Table S1: Blast analysis of the partial 16S rRNA gene sequence of *Lactobacillus* SP3.
