# Supplementary material for: Application of A Novel Potential Probiotic Lactobacillus paracasei Strain Isolated from Kefir Grains in the Production of Feta-Type Cheese
Source: Microorganisms. 2018 Nov 29;6(4):121. doi: 10.3390/microorganisms6040121 (PMC6313735; doi:10.3390/microorganisms6040121)
Supplement: Supplementary file 1 [file microorganisms-06-00121-s001.pdf]

Table S1. Blast analysis of the partial 16S rRNA gene sequence of *Lactobacillus* SP3.

***Lactobacillus* strain**	**Identity**	**Accession number**
Lactobacillus paracasei strain LL13	99%	KX951732.1
Lactobacillus paracasei strain RCM2	99%	KT626389.1
Lactobacillus casei strain ZT-Lca.62	99%	KM921936.1
Lactobacillus casei strain ZT-Lca.65	99%	KM921935.1
Lactobacillus casei strain OC6	99%	JN851813.1
Lactobacillus paracasei strain CACIO12CT	99%	KU315107.1
Lactobacillus paracasei strain CB8CT	99%	KU315101.1
Lactobacillus paracasei strain UP2F	99%	MK071312.1
Lactobacillus paracasei strain MA10-5	99%	MG755370.1
Lactobacillus casei strain L25	99%	MH094125.1
Lactobacillus casei strain L20	99%	MH094124.1
Lactobacillus casei strain 111	99%	MH997808.1
Lactobacillus casei strain ACP01	99%	MK032218.1
Lactobacillus casei strain P5-1	99%	MK024231.1
Lactobacillus paracasei strain 4G330	99%	MK026811.1
